# Supplementary figures and images for: Identification of key genes in non-small cell lung cancer by bioinformatics analysis
Source: PeerJ. 2019 Dec 12;7:e8215. doi: 10.7717/peerj.8215 (PMC6911687; doi:10.7717/peerj.8215)

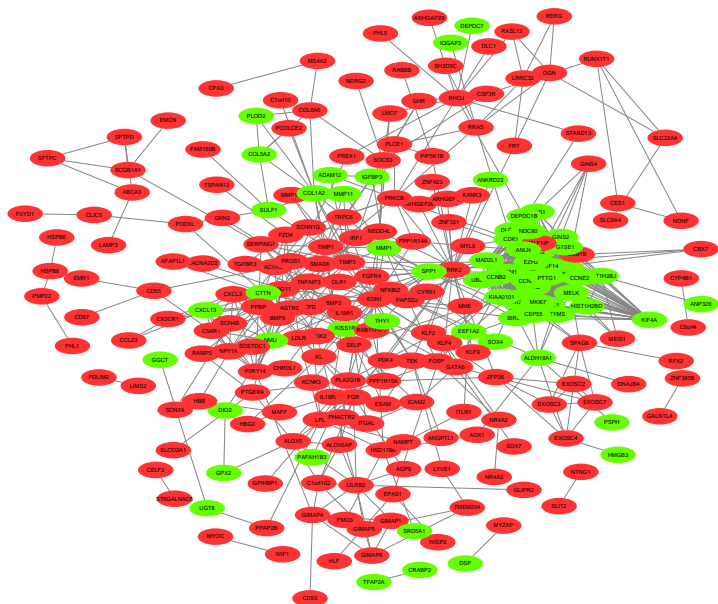

Supplement: Figure S1 — The PPI network of 685 DEGs was constructed from STRING to predict the interactions of identified DEGs, consisting of 249 nodes and 1027 edges.The up-regulated genes were in red and the down-regulated genes were in green. [file peerj-07-8215-s001.pdf]

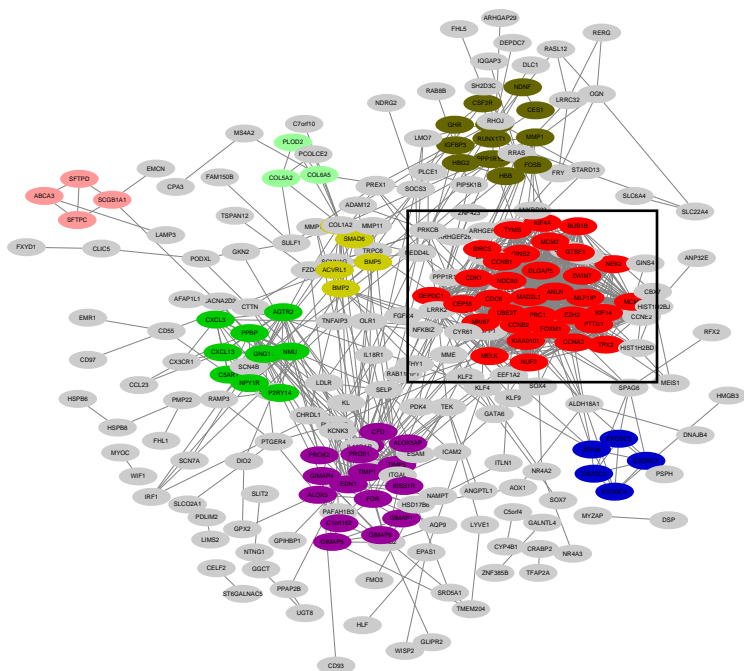

Supplement: Figure S2 — The eight functional modules of PPI network were screened out by Cytoscape’s app MCODE. Among these eight modules, module 1 was found to be located in the center of the entire PPI network including 33 nodes and 510 edges and it was showed in the black box. [file peerj-07-8215-s002.pdf]
