# Supplementary material for: Identification of key genes in non-small cell lung cancer by bioinformatics analysis
Source: PeerJ. 2019 Dec 12;7:e8215. doi: 10.7717/peerj.8215 (PMC6911687; doi:10.7717/peerj.8215)
Supplement: Table S1 [file peerj-07-8215-s003.docx]

Supplementary Table.1 DEGs in the identification of three datasets

| Dataset | DEGs |
| --- | --- |
| GSE33532 | 14373 |
| GSE30219 | 21740 |
| GSE19804 | 20748 |
