# Supplementary material for: Identification of key genes in non-small cell lung cancer by bioinformatics analysis
Source: PeerJ. 2019 Dec 12;7:e8215. doi: 10.7717/peerj.8215 (PMC6911687; doi:10.7717/peerj.8215)
Supplement: Table S2 [file peerj-07-8215-s004.docx]

| Supplementary Table. 2 GO Enrichment Analysis for genes in module 1 | | | | |
| --- | --- | --- | --- | --- |
| Category | Term | Gene Count | PValue | Gene name |
| GOTERM_BP_DIRECT | GO:0051301~cell division | 15 | 4.24E-16 | KIF14, CDC6, CDK1, NEK2, NUF2, TPX2, NDC80, BIRC5, PTTG1, CCNB1, CCNB2, MAD2L1, ZWINT, BUB1B, CCNA2 |
| GOTERM_BP_DIRECT | GO:0007067~mitotic nuclear division | 13 | 9.12E-15 | CDC6, CDK1, NEK2, NUF2, TPX2, ANLN, BIRC5, NDC80, PTTG1, CEP55, CCNB2, BUB1B, CCNA2 |
| GOTERM_CC_DIRECT | GO:0005634~nucleus | 27 | 6.03E-10 | PRC1, NEK2, FOXM1, EZH2, KIAA0101, PTTG1, TYMS, CCNA2, KIF14, GINS2, CDC6, CDK1, MKI67, DLGAP5, TPX2, NUF2, NDC80, BIRC5, DEPDC1, MCM2, MCM4, CCNB1, MAD2L1, CCNB2, ZWINT, MELK, UBE2T |
| GOTERM_BP_DIRECT | GO:0000086~G2/M transition of mitotic cell cycle | 8 | 4.61E-09 | CCNB1, CDK1, CCNB2, NEK2, FOXM1, TPX2, BIRC5, MELK |
| GOTERM_CC_DIRECT | GO:0005654~nucleoplasm | 20 | 6.51E-09 | CDC6, GINS2, CDK1, KIF4A, PRC1, FOXM1, EZH2, TPX2, KIAA0101, BIRC5, ANLN, DEPDC1, MCM2, MCM4, GTSE1, CCNB1, TYMS, CCNB2, CCNA2, UBE2T |
| GOTERM_CC_DIRECT | GO:0000777~condensed chromosome kinetochore | 7 | 6.65E-09 | MAD2L1, NEK2, ZWINT, NUF2, BUB1B, BIRC5, NDC80 |
| GOTERM_CC_DIRECT | GO:0030496~midbody | 7 | 7.13E-08 | KIF14, CDK1, KIF4A, PRC1, NEK2, BIRC5, CEP55 |
| GOTERM_CC_DIRECT | GO:0000922~spindle pole | 6 | 1.05E-06 | CCNB1, CDC6, MAD2L1, PRC1, NEK2, TPX2 |
| GOTERM_BP_DIRECT | GO:0007062~sister chromatid cohesion | 6 | 1.18E-06 | MAD2L1, ZWINT, NUF2, BUB1B, BIRC5, NDC80 |
| GOTERM_CC_DIRECT | GO:0005829~cytosol | 18 | 4.91E-06 | KIF14, CDC6, CDK1, KIF4A, PRC1, NEK2, NUF2, TPX2, NDC80, BIRC5, PTTG1, GTSE1, CCNB1, TYMS, MAD2L1, CCNB2, ZWINT, BUB1B |
| GOTERM_MF_DIRECT | GO:0005515~protein binding | 29 | 6.24E-06 | KIF4A, PRC1, NEK2, FOXM1, EZH2, KIAA0101, PTTG1, CEP55, GTSE1, CCNA2, KIF14, GINS2, CDC6, CDK1, MKI67, DLGAP5, TPX2, NUF2, NDC80, BIRC5, DEPDC1, MCM2, MCM4, CCNB1, MAD2L1, CCNB2, ZWINT, BUB1B, MELK |
| GOTERM_BP_DIRECT | GO:0006260~DNA replication | 6 | 8.79E-06 | CDK1, GINS2, CDC6, KIAA0101, MCM2, MCM4 |
| GOTERM_CC_DIRECT | GO:0000776~kinetochore | 5 | 1.04E-05 | MAD2L1, NEK2, ZWINT, BUB1B, NDC80 |
| GOTERM_BP_DIRECT | GO:0000070~mitotic sister chromatid segregation | 4 | 1.27E-05 | MAD2L1, NEK2, ZWINT, NDC80 |
| GOTERM_BP_DIRECT | GO:0031145~anaphase-promoting complex-dependent catabolic process | 5 | 1.30E-05 | CCNB1, CDK1, MAD2L1, BUB1B, PTTG1 |
| GOTERM_BP_DIRECT | GO:0000082~G1/S transition of mitotic cell cycle | 5 | 3.56E-05 | CDK1, CDC6, TYMS, MCM2, MCM4 |
| GOTERM_MF_DIRECT | GO:0005524~ATP binding | 12 | 4.05E-05 | KIF14, CDK1, CDC6, KIF4A, MKI67, NEK2, TPX2, BUB1B, MCM2, MCM4, UBE2T, MELK |
| GOTERM_BP_DIRECT | GO:0008283~cell proliferation | 7 | 4.78E-05 | CDK1, TYMS, MKI67, DLGAP5, TPX2, BUB1B, MELK |
| GOTERM_CC_DIRECT | GO:0005876~spindle microtubule | 4 | 5.63E-05 | CDK1, KIF4A, PRC1, BIRC5 |
| GOTERM_CC_DIRECT | GO:0000775~chromosome, centromeric region | 4 | 1.23E-04 | MKI67, NUF2, BIRC5, NDC80 |
| GOTERM_BP_DIRECT | GO:0042787~protein ubiquitination involved in ubiquitin-dependent protein catabolic process | 5 | 1.72E-04 | CCNB1, CDK1, MAD2L1, BUB1B, PTTG1 |
| GOTERM_BP_DIRECT | GO:0051436~negative regulation of ubiquitin-protein ligase activity involved in mitotic cell cycle | 4 | 2.99E-04 | CCNB1, CDK1, MAD2L1, BUB1B |
| GOTERM_BP_DIRECT | GO:0051437~positive regulation of ubiquitin-protein ligase activity involved in regulation of mitotic cell cycle transition | 4 | 3.66E-04 | CCNB1, CDK1, MAD2L1, BUB1B |
| GOTERM_CC_DIRECT | GO:0051233~spindle midzone | 3 | 4.70E-04 | KIF14, CDC6, BUB1B |
| GOTERM_BP_DIRECT | GO:0043161~proteasome-mediated ubiquitin-dependent protein catabolic process | 5 | 5.05E-04 | KIF14, CDK1, MAD2L1, BUB1B, GTSE1 |
| GOTERM_MF_DIRECT | GO:0019901~protein kinase binding | 6 | 5.63E-04 | KIF14, CCNB1, PRC1, FOXM1, TPX2, CCNA2 |
| GOTERM_CC_DIRECT | GO:0005737~cytoplasm | 19 | 5.83E-04 | CDC6, CDK1, KIF4A, MKI67, PRC1, NEK2, DLGAP5, FOXM1, EZH2, KIAA0101, BIRC5, PTTG1, MCM2, CCNB1, TYMS, ZWINT, BUB1B, CCNA2, UBE2T |
| GOTERM_CC_DIRECT | GO:0016020~membrane | 12 | 6.50E-04 | KIF14, CCNB1, CDK1, KIF4A, CCNB2, MKI67, NUF2, NDC80, CEP55, MCM4, GTSE1, MELK |
| GOTERM_BP_DIRECT | GO:0000083~regulation of transcription involved in G1/S transition of mitotic cell cycle | 3 | 8.15E-04 | CDK1, CDC6, TYMS |
| GOTERM_BP_DIRECT | GO:0045931~positive regulation of mitotic cell cycle | 3 | 0.001210048 | CCNB1, CDK1, BIRC5 |
| GOTERM_BP_DIRECT | GO:0000281~mitotic cytokinesis | 3 | 0.001298188 | KIF4A, ANLN, CEP55 |
| GOTERM_BP_DIRECT | GO:0051726~regulation of cell cycle | 4 | 0.001519019 | CCNB1, CCNB2, FOXM1, KIAA0101 |
| GOTERM_BP_DIRECT | GO:0007093~mitotic cell cycle checkpoint | 3 | 0.001580507 | MAD2L1, ZWINT, BUB1B |
| GOTERM_BP_DIRECT | GO:0006270~DNA replication initiation | 3 | 0.001580507 | CDC6, MCM2, MCM4 |
| GOTERM_CC_DIRECT | GO:0015630~microtubule cytoskeleton | 4 | 0.001601428 | CCNB2, PRC1, TPX2, MCM2 |
| GOTERM_MF_DIRECT | GO:0004693~cyclin-dependent protein serine/threonine kinase activity | 3 | 0.001765113 | CCNB1, CDK1, CCNB2 |
| GOTERM_CC_DIRECT | GO:0005874~microtubule | 5 | 0.001819752 | KIF14, KIF4A, NEK2, TPX2, BIRC5 |
| GOTERM_BP_DIRECT | GO:0090307~mitotic spindle assembly | 3 | 0.001998297 | NEK2, TPX2, BIRC5 |
| GOTERM_BP_DIRECT | GO:0007080~mitotic metaphase plate congression | 3 | 0.002110061 | KIF14, CCNB1, CEP55 |
| GOTERM_BP_DIRECT | GO:0007077~mitotic nuclear envelope disassembly | 3 | 0.002973191 | CCNB1, CDK1, CCNB2 |
| GOTERM_BP_DIRECT | GO:0031100~organ regeneration | 3 | 0.003385811 | CDK1, MKI67, CCNA2 |
| GOTERM_BP_DIRECT | GO:0006915~apoptotic process | 6 | 0.003539832 | CDK1, TPX2, BUB1B, BIRC5, MCM2, MELK |
| GOTERM_BP_DIRECT | GO:0048146~positive regulation of fibroblast proliferation | 3 | 0.004446221 | CCNB1, CDC6, CCNA2 |
| GOTERM_CC_DIRECT | GO:0005813~centrosome | 5 | 0.005620891 | CCNB1, CDK1, CCNB2, NEK2, CEP55 |
| GOTERM_BP_DIRECT | GO:0006977~DNA damage response, signal transduction by p53 class mediator resulting in cell cycle arrest | 3 | 0.00582187 | CCNB1, CDK1, GTSE1 |
| GOTERM_MF_DIRECT | GO:0008017~microtubule binding | 4 | 0.006428413 | KIF14, KIF4A, PRC1, BIRC5 |
| GOTERM_CC_DIRECT | GO:0000942~condensed nuclear chromosome outer kinetochore | 2 | 0.00678743 | CCNB1, NDC80 |
| GOTERM_CC_DIRECT | GO:0031262~Ndc80 complex | 2 | 0.00678743 | NUF2, NDC80 |
| GOTERM_BP_DIRECT | GO:0007059~chromosome segregation | 3 | 0.006965327 | NEK2, NUF2, NDC80 |
| GOTERM_MF_DIRECT | GO:0035173~histone kinase activity | 2 | 0.007325977 | CCNB1, CDK1 |
| GOTERM_BP_DIRECT | GO:0006281~DNA repair | 4 | 0.009111696 | CDK1, FOXM1, PTTG1, UBE2T |
| GOTERM_BP_DIRECT | GO:0021695~cerebellar cortex development | 2 | 0.011027341 | KIF14, EZH2 |
| GOTERM_BP_DIRECT | GO:0090267~positive regulation of mitotic cell cycle spindle assembly checkpoint | 2 | 0.012853753 | MAD2L1, NDC80 |
| GOTERM_CC_DIRECT | GO:0042555~MCM complex | 2 | 0.015209041 | MCM2, MCM4 |
| GOTERM_CC_DIRECT | GO:0005819~spindle | 3 | 0.017930691 | PRC1, TPX2, BIRC5 |
| GOTERM_BP_DIRECT | GO:0055015~ventricular cardiac muscle cell development | 2 | 0.018313425 | CCNB1, CDK1 |
| GOTERM_BP_DIRECT | GO:0006268~DNA unwinding involved in DNA replication | 2 | 0.018313425 | MCM2, MCM4 |
| GOTERM_BP_DIRECT | GO:0034501~protein localization to kinetochore | 2 | 0.018313425 | CDK1, BUB1B |
| GOTERM_CC_DIRECT | GO:0000784~nuclear chromosome, telomeric region | 3 | 0.020514579 | CDK1, MCM2, MCM4 |
| GOTERM_BP_DIRECT | GO:0007095~mitotic G2 DNA damage checkpoint | 2 | 0.027348005 | CDK1, CCNA2 |
| GOTERM_MF_DIRECT | GO:0004672~protein kinase activity | 4 | 0.027615171 | CDK1, NEK2, BUB1B, MELK |
| GOTERM_MF_DIRECT | GO:0048037~cofactor binding | 2 | 0.028993623 | TYMS, BIRC5 |
| GOTERM_MF_DIRECT | GO:0008574~ATP-dependent microtubule motor activity, plus-end-directed | 2 | 0.030778455 | KIF14, KIF4A |
| GOTERM_MF_DIRECT | GO:0004674~protein serine/threonine kinase activity | 4 | 0.031084536 | CDK1, NEK2, BUB1B, MELK |
| GOTERM_MF_DIRECT | GO:0003682~chromatin binding | 4 | 0.034329491 | CDK1, EZH2, KIAA0101, UBE2T |
| GOTERM_BP_DIRECT | GO:0007094~mitotic spindle assembly checkpoint | 2 | 0.036302096 | MAD2L1, BUB1B |
| GOTERM_BP_DIRECT | GO:1900182~positive regulation of protein localization to nucleus | 2 | 0.038083317 | CDK1, GTSE1 |
| GOTERM_BP_DIRECT | GO:0060045~positive regulation of cardiac muscle cell proliferation | 2 | 0.039861351 | CCNB1, CDK1 |
| GOTERM_BP_DIRECT | GO:0051439~regulation of ubiquitin-protein ligase activity involved in mitotic cell cycle | 2 | 0.041636205 | CCNB1, CDK1 |
| GOTERM_MF_DIRECT | GO:0003678~DNA helicase activity | 2 | 0.043183684 | MCM2, MCM4 |
| GOTERM_BP_DIRECT | GO:0007088~regulation of mitotic nuclear division | 2 | 0.043407884 | MKI67, NEK2 |
| GOTERM_BP_DIRECT | GO:0043066~negative regulation of apoptotic process | 4 | 0.050733414 | KIF14, CDK1, MAD2L1, BIRC5 |
| GOTERM_BP_DIRECT | GO:0097421~liver regeneration | 2 | 0.05221884 | TYMS, EZH2 |
| GOTERM_BP_DIRECT | GO:0008284~positive regulation of cell proliferation | 4 | 0.053781076 | KIF14, PRC1, FOXM1, BIRC5 |
| GOTERM_BP_DIRECT | GO:0007052~mitotic spindle organization | 2 | 0.053971582 | CCNB1, NDC80 |
| GOTERM_MF_DIRECT | GO:0004869~cysteine-type endopeptidase inhibitor activity | 2 | 0.060639242 | BIRC5, PTTG1 |
| GOTERM_BP_DIRECT | GO:0045892~negative regulation of transcription, DNA-templated | 4 | 0.063447241 | FOXM1, EZH2, BIRC5, DEPDC1 |
| GOTERM_BP_DIRECT | GO:0032467~positive regulation of cytokinesis | 2 | 0.066153244 | KIF14, CDC6 |
| GOTERM_CC_DIRECT | GO:0072686~mitotic spindle | 2 | 0.067493695 | CDK1, MAD2L1 |
| GOTERM_BP_DIRECT | GO:0000079~regulation of cyclin-dependent protein serine/threonine kinase activity | 2 | 0.069605745 | CDC6, CCNA2 |
| GOTERM_BP_DIRECT | GO:0045184~establishment of protein localization | 2 | 0.071327361 | KIF14, CEP55 |
| GOTERM_MF_DIRECT | GO:0015631~tubulin binding | 2 | 0.074381417 | KIF14, BIRC5 |
| GOTERM_BP_DIRECT | GO:0032147~activation of protein kinase activity | 2 | 0.079889297 | KIF14, TPX2 |
| GOTERM_CC_DIRECT | GO:0005881~cytoplasmic microtubule | 2 | 0.083274352 | BIRC5, GTSE1 |
| GOTERM_BP_DIRECT | GO:0000910~cytokinesis | 2 | 0.084989738 | PRC1, BIRC5 |
| GOTERM_CC_DIRECT | GO:0005871~kinesin complex | 2 | 0.086399321 | KIF14, KIF4A |
| GOTERM_BP_DIRECT | GO:0014070~response to organic cyclic compound | 2 | 0.086683797 | CDK1, MKI67 |
| GOTERM_BP_DIRECT | GO:0021766~hippocampus development | 2 | 0.09845751 | KIF14, EZH2 |
